# Comparative characteristics of early-onset vs. late-onset advanced colorectal cancer: a nationwide study in China

**DOI:** 10.1186/s12885-024-12278-7

**Published:** 2024-04-20

**Authors:** Hongwei Liu, Huifang Xu, Yin Liu, Yuqian Zhao, Xi Zhang, Yanqin Yu, Lingbin Du, Yunyong Liu, Wenjun Wang, Helu Cao, Li Ma, Juanxiu Huang, Ji Cao, Li Li, Yanping Fan, Xiaofen Gu, Changyan Feng, Qian Zhu, Xiaohui Wang, Jingchang Du, Shaokai Zhang, Youlin Qiao

**Affiliations:** 1 Department of Cancer Epidemiology, The Affiliated Cancer Hospital of Zhengzhou University & Henan Cancer Hospital, Zhengzhou, 450008 China; 2 Office of Academic Research, Sichuan Clinical Research Center for Cancer, Sichuan Cancer Hospital & Institute, Sichuan Cancer Center, Affiliated Cancer Hospital of University of Electronic Science and Technology of China, Chengdu, China; 3 Key Laboratory of Carcinogenesis and Translational Research (Ministry of Education/Beijing), Beijing Office for Cancer Prevention and Control, Peking University Cancer Hospital and Institute, Beijing, China; 4 The Clinical Epidemiology of Research Center, Department of Dermatological, The First Affiliated Hospital of Baotou Medical College, Baotou, China; 5 Department of Cancer Prevention, The Cancer Hospital of the University of Chinese Academy of Sciences, Zhejiang Cancer Hospital, Hangzhou, China; 6 Office of Shenzhen Cancer Prevention and Control Center, National Cancer Center/National Clinical Research Center for Cancer/Cancer Hospital & Shenzhen Hospital, Chinese Academy of Medical Sciences and Peking Union Medical College, Shenzhen, China; 7 School of Nursing, Jining Medical University, Jining, China; 8 Department of Preventive Health, Xinxiang Central Hospital, Xinxiang, China; 9 Public Health School, Dalian Medical University, Dalian, China; 10 Department of Gastroenterology, Wuzhou Red Cross Hospital, Wuzhou, China; 11 Department of Cancer Prevention and Control Office, The First Affiliated Hospital of Guangxi Medical University, Nanning, Guangxi China; 12 Department of Clinical Research, The First Affiliated Hospital, Jinan University, Guangzhou, China; 13 State Key Laboratory of Oncology in South China, Collaborative Innovation Center for Cancer Medicine, Sun Yat-sen University Cancer Center, Guangzhou, China; 14 Department of Student Affairs, Affiliated Tumor Hospital, Xinjiang Medical University, Ürümqi, Xinjiang China; 15 Chongqing Key Laboratory of Translational Research for Cancer Metastasis and Individualized Treatment, Chongqing University Cancer Hospital, Chongqing, China; 16 School of Public Health and Management, Chongqing Medical University, Chongqing, China; 17 Department of Public Health, Gansu Provincial Cancer Hospital, Lanzhou, China; 18 School of Public Health, Chengdu Medical College, Chengdu, China; 19 Center for Global Health, School of Population Medicine and Public Health, Chinese Academy of Medical Sciences and Peking Union Medical College, Beijing, 100005 China

**Keywords:** Early-onset colorectal cancer, Late-onset colorectal cancer, Clinical epidemiology features, Health-related quality of life

## Abstract

**Background:**

The incidence of early-onset colorectal cancer (EOCRC, diagnosed in patients under the age of 50 years) has been increasing around the world. Here, we aimed to systematically identify distinctive features of EOCRC.

**Methods:**

From 2020 to 2021, we conducted a nationwide survey in 19 hospitals, collecting data on advanced CRC patients’ demographics, clinical features, disease knowledge, medical experiences, expenditures, and health-related quality of life (HRQOL). We compared these features between EOCRC and late-onset colorectal cancer (LOCRC, ≥ 50 years old) groups and analyzed the association between EOCRC and HRQOL using multivariate linear regression.

**Findings:**

In total, 991 patients with EOCRC and 3581 patients with LOCRC were included. Compared to the LOCRC group, the EOCRC group had higher levels of education, were more informed about the risk factors for CRC, were more likely to have widespread metastases throughout the body, were more inclined to undergo gene testing, and were more likely to opt for targeted therapy, radiotherapy, and chemotherapy. However, HRQOL in the EOCRC group was similar to that of the LOCRC group, and no significant association was observed between EOCRC and HRQOL (beta: -0.753, *P* value: 0.307).

**Interpretation:**

In Chinese patients, EOCRC patients had more aggressive features. Despite undergoing more intensified treatments and gene testing, they had similar HRQOL compared with LOCRC. These findings advocate for a more tailored approach to treatment, especially for young CRC patients with advanced TNM stages and metastasis.

**Supplementary Information:**

The online version contains supplementary material available at 10.1186/s12885-024-12278-7.

## Introduction

Colorectal cancer (CRC) is the third most common cancer worldwide and the second leading cause of cancer-related mortality [1]. Based on the age at diagnosis, CRC can be classified as the early-onset colorectal cancer (EOCRC), which is diagnosed before 50 years of age, and the late-onset colorectal cancer (LOCRC), which is diagnosed at or after 50 years of age [2]. The LOCRC incidence has generally decreased since the early 1990s [1], especially in western countries, which is likely attributable to population-based screening with colonoscopy. In contrast, the incidence of EOCRC has steadily increased by 2% annually [3–5]. The reasons for this increase in EOCRC, including potential unique biological characteristics compared to LOCRC, remain unclear.

Multiple studies have demonstrated that EOCRC are more likely to exhibit symptoms, including haematochezia and abdominal pain, to occur in the left colon, have more aggressive histopathology, and have a longer delay from symptom onset to diagnosis [2–6] Regarding the treatment patterns, the EOCRC group has more intensified surgical and perioperative treatment than the LOCRC group [7]. It is reported that the mutations of *PIK3CA*, *BRAF*, and *KRAS* were different between EOCRC and LOCRC patients according to different tumor locations [8–10]. To date, differences between EOCRC and LOCRC have been primarily investigated in developed Western countries. However, nationwide studies among EOCRC patients in China are generally lacking, especially regarding the demographic and clinicopathological features, awareness of CRC, treatment, and health-related quality of life (HRQOL) in EOCRC patients.

To provide a clearer landscape of the characteristics of EOCRC in Chinese patients, we conduct a nationwide survey, comparing the demographic, clinicopathological features, disease knowledge, treatment, and HRQOL, aimed at identity the characteristics of the EOCRC and LOCRC among CRC patients in China.

## Methods

### Study design

**Fig. 1:**
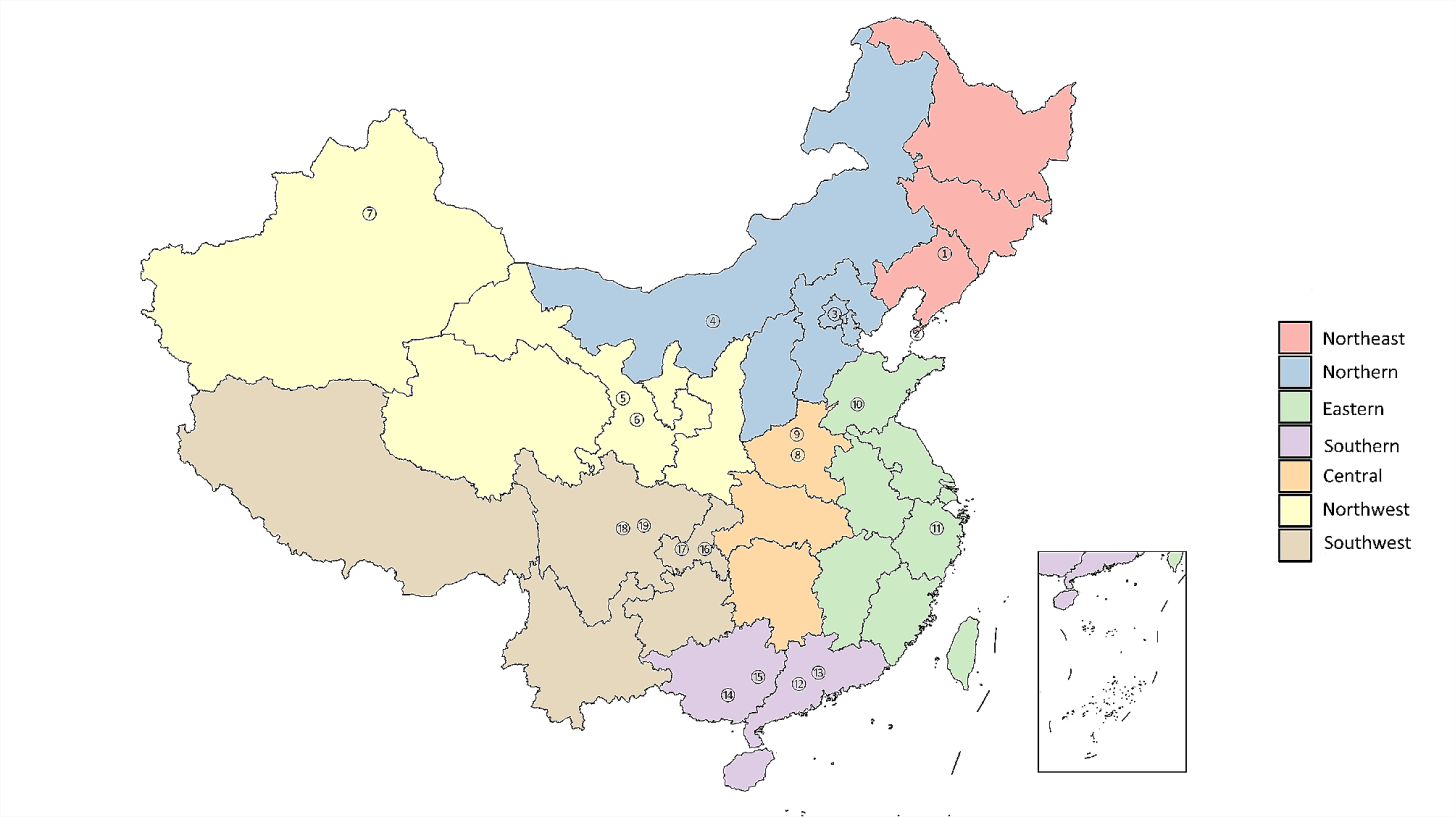
Map of the 19 hospitals and geographical regions in China. *Note* The corresponding hospitals for each number are detailed in Table S1

This is a nationwide multicenter cross-sectional survey and the comprehensive study design has been previously published [11]. The study was conducted from March 2020 to March 2021. Advanced colorectal cancer patients were sampled using a multi-stage sampling method. In the first stage, two cities of each geographic regions (Eastern China, Northern China, Central China, Southern China, Northeast China, Southwest China, and Northwest China) of Chinese mainland were selected by simple random sampling. In the second stage, one tertiary cancer hospital and/or one general hospital were selected in each city with inclusion on the basis that (1) they can provide diagnosis, surgery, radiotherapy, chemotherapy and routine follow- up care for patients with CRC; and (2) visiting patients are from different parts of the region. Finally, a total of 19 hospitals were selected. The detailed information on enrollment data for 19 hospitals from 7 regions is presented in Fig. 1 and Table S1.

In the present study, the primary objective is to compare the demographic, clinical features, disease knowledge, and HRQOL between EOCRC and LOCRC patients. For secondary objectives, we delve into the treatment patterns, factors influencing HRQOL, and the variations in disease management practices between EOCRC and LOCRC patients.

### Inclusion and exclusion criteria

Patients who met the following inclusion criteria were included: (1) are diagnosed with stage III or IV CRC at the survey, (2) are aged ≥ 18 years old, (3) are inpatients and (4) provide the informed consent. Patients will be excluded if they had severe physical, cognitive and/or verbal impairments that would interfere with a patient’s ability to complete the questionnaire.

## Measurements

### Demographic and clinicopathologic characteristics

Demographic data were collected through a standardized self-report questionnaire, including age at the first diagnosis of CRC, gender, marital status, education level, geographic region, and occupation. Clinicopathological characteristics included the site of cancer occurrence (colon or rectum), pathological TNM stage at first diagnosis, metastatic status at the survey, the reason for the initial hospital visit, and the number of hospitals visited.

### Awareness of CRC risk factors, screening, and treatment

Patients’ awareness regarding high-risk factors for colorectal cancer, CRC screening procedures, and treatment options before their diagnosis was gathered through a semi-structured questionnaire (SSQ). The SSQ was developed following the Chinese guidelines [12, 13]. It comprises three multiple-choice questions, and detailed information on the questions is presented in Table S2. Further details about the SSQ can be found in a previously published study [11].

### Patients’ experiences with CRC screening, diagnosis, and treatment

Another SSQ was employed to gather information concerning CRC screening, diagnosis, and treatment. Patient screening history data were collected, including whether the patient had undergone screening, and information about barriers to not having a colonoscopy was collected based on patient self-reports. These barriers included lack of awareness, insufficient time for a colonoscopy, concerns about the discomfort associated with the procedure, cost-related challenges, waiting time for colonoscopy appointments, and issues with insurance coverage. With regards to CRC diagnosis and treatment, the following information was collected based on patients’ self-reports: (1) the utilization of gene testing, any barriers encountered, and the results of gene testing. (2) the adoption of currently available treatment modalities, such as targeted therapy, surgery, radiotherapy, chemotherapy, endoscopic treatment, and immunotherapy.

### Medical expenditure

Medical expenses data was collected either from the medical records, or through patients’ self-reports. The gathered information will encompass patients’ out-of-pocket expenditures related to CRC diagnosis and treatment, reimbursement rates for all medical costs, annual household income, the perspective of patients on the cost of colorectal cancer treatment, and the type of health insurance.

### Health-related quality of life

Health-related quality of life (HRQOL) was assessed based on two questionnaires: the Chinese Functional Assessment of Cancer Therapy-Colorectal (FACT-C) V.4 and the Chinese version of the European Organization for Research and Treatment of Cancer (EORTC) QLQ-C30 V.3. The FACT-C V.4 comprises 36 items distributed across five function subscales: physical, social/family, emotional, functional, and a colorectal cancer subscale [11, 14, 15]. Meanwhile, the traditional Chinese version of EORTC QLQ-C30 V.3 includes 30 items grouped into five function subscales (physical, role, emotional, cognitive, and social), nine symptom subscales (fatigue, nausea/vomiting, pain, dyspnea, insomnia, appetite loss, constipation, diarrhoea, and financial difficulties), and a global health/QOL subscale [16, 17]. In this study, a scale named FACT-C-plus-QLQ-C9 was created, consisting of 45 items selected based on expert opinions. This scale includes all FACT-C items along with nine items from QLQ-C30, as outlined in Table S3. The self-developed scale covers six functioning subscales (physical, social/family, emotional, functional, colorectal cancer subscale, and cognitive), two symptom subscales (fatigue and sleep disturbance), and one item related to financial difficulties. Higher scores on the functioning subscales and lower scores on the symptom subscales indicate a better quality of life. The Chinese versions of FACT-C and EORTC QLQ-C30 have been validated in prior studies [14–17].

Patients’ quality of life was assessed after CRC treatment. The summary score of HRQOL for each patient will be calculated across all items, including functioning scales and symptom scales (with inverted scores), resulting in a range from 0 to 180. A higher score indicates a better HRQOL. The Cronbach’s α coefficient of HRQOL in our questionnaire was 0.80.

### Statistical analysis

Categorical data were described by frequencies and percentages, and continuous data using standard deviations (SD). The *t*-test, chi-square test, and Mann–Whitney U test were used to compare the characteristics of the EOCRC and LOCRC groups. Multivariate regressions were conducted to evaluate the associations between early diagnosis and HRQOL. The following variables were adjusted in multivariate regressions: HRQOL before treatment, sex, cancer location, education level, and TNM stage at initial diagnosis. Statistical significance was set at a *P*-value < 0.05. Data analysis was performed using R software (version 4.2.0, R Foundation for Statistical Computing).

## Results

A total of 4572 cases of CRC were included in this study. A flowchart of the patient selection process is shown in Fig. S1. Of the included patients, 59.5% were men; 54.5% had rectal cancer; and 37.5% had metastasis. The age distribution at the time of diagnosis is presented in Fig. S2, and the median age at diagnosis was 59.42 years. Patients were classified into two groups based on their age at diagnosis: EOCRC, < 50 years (*N* = 991), and LOCRC, ≥ 50 years (*N* = 3581).

Clinicopathologic characteristics of patients with EOCRC and LOCRC are summarized in Table 1. In terms of education level, the EOCRC group had a higher percentage of patients with a university/specialty degree or above (EOCRC = 27.9%; LOCRC = 12.7%) and a lower percentage of those with primary school education or below (EOCRC = 16.2%; LOCRC = 32.5%) than that in the LOCRC group. Additionally, the patients with EOCRC exhibited a higher prevalence of widespread metastases (EOCRC = 19.7%; LOCRC = 14.1%) and a greater frequency of hospital visits (EOCRC = 2.10; LOCRC = 1.89). However, there were no statistically significant differences among the two groups concerning the location of cancer occurrence, the TNM stage at the time of the first diagnosis, or the reasons for the initial hospital visit.

**Table 1:** Clinicopathologic characteristics of patients with early-onset and late-onset colorectal cancer

	Overall (*N* = 4572)	EOCRC, < 50 years (*N* = 991)	LOCRC, ≥ 50 years (*N* = 3581)	*P* value
**Age at diagnosis, years**	58.64 (11.71)	42.24 (6.85)	63.18 (8.20)	< 0.001
**Sex**				0.001
Male	2720 (59.5)	544 (54.9)	2176 (60.8)	
Female	1852 (40.5)	447 (45.1)	1405 (39.2)	
**Marital status**				0.001
Married	4318 (94.1)	911 (91.9)	3390 (94.7)	
Not married/divorced/widowed	270 (5.9)	80 (8.1)	190 (5.3)	
**Education level**				< 0.001
Primary school or below	1325 (29.0)	161 (16.2)	1164 (32.5)	
Middle school	1475 (32.3)	343 (34.6)	1132 (31.6)	
High school/specialized secondary schools	1037 (22.7)	211 (21.3)	826 (23.1)	
University/specialty or above	732 (16.0)	276 (27.9)	456 (12.7)	
**Region**				< 0.001
Eastern	1312 (28.7)	233 (23.5)	1079 (30.1)	
Northern	563 (12.3)	112 (11.3)	451 (12.6)	
Southern	665 (14.5)	197 (19.9)	468 (13.1)	
Central	689 (15.1)	141 (14.2)	548 (15.3)	
Northeast	364 (8.0)	60 (6.1)	304 (8.5)	
Southwest	652 (14.3)	165 (16.6)	487 (13.6)	
Northwest	327 (7.2)	83 (8.4)	244 (6.8)	
**Occupation**				< 0.001
Government and public sector personnel	653 (14.3)	271 (27.3)	382 (10.7)	
Service workers, migrant workers, and individuals	1726 (37.8)	472 (47.6)	1254 (35.0)	
Unemployment, layoffs, etc.	1929 (42.2)	181 (18.3)	1748 (48.8)	
Unknow	264 (5.8)	67 (6.8)	197 (5.5)	
**Location of cancer**				0.107
Colon	2054 (45.5)	83 (53.5)	1587 (44.8)	
Rectum	2463 (54.5)	72 (46.5)	1953 (55.2)	
**Pathological TNM stage at first diagnosis**				0.032
I	110 (2.5)	19 (2.0)	91 (2.7)	
II	772 (17.6)	141 (14.7)	631 (18.4)	
III	1964 (44.7)	446 (46.6)	1518 (44.2)	
IV	1545 (35.2)	352 (36.7)	1193 (34.8)	
**Metastasis at first diagnosis**				< 0.001
No metastasis	2842 (62.5)	598 (60.9)	2244 (63.0)	
With liver metastasis only	639 (14.1)	126 (12.8)	513 (14.4)	
With lung metastasis only	179 (3.9)	34 (3.5)	145 (4.1)	
With both liver and lung metastases	191 (4.2)	31 (3.2)	160 (4.5)	
Widespread metastases throughout the body	695 (15.3)	193 (19.7)	502 (14.1)	
**Reason for the first hospital visit**				0.548
Observation of suspected symptoms by patients themselves	4003 (88.1)	882 (89.2)	3121 (87.7)	
Physical examination findings	265 (5.8)	51 (5.2)	214 (6.0)	
Detection of CRC during screening or treatment of other diseases	278 (6.1)	56 (5.7)	222 (6.2)	
**Number of visited hospital**	1.94 (0.81)	2.10 (0.83)	1.89 (0.80)	< 0.001

*Abbreviations* EOCRC, Early-onset colorectal cancer; LOCRC, Late-onset colorectal cancer

Values are presented as mean (standard deviations) for continuous variables or n (%) for categorical variables

Table 2 summarizes the characteristics of disease knowledge, medical experience, and expenditure. Compared with the LOCRC group, the EOCRC group had a larger proportion of patients knowing about CRC risk factors and screening, although this difference was not statistically significant. Additionally, more EOCRC patients underwent gene testing (EOCRC = 56.7%; LOCRC = 44.4%; *P* < 0.001). There were no significant differences in *RAS*, *BRAF* mutations, or microsatellite instability (MSI) among two groups. Regarding the treatment modalities, the EOCRC group exhibited a higher percentage of patients opting for targeted therapy, radiotherapy, and chemotherapy. In terms of medical expenditure, there were significant differences in the out-of-pocket medical expenditure, medical expenditure reimbursement ratio, annual household income, and health insurance among the two groups.

**Table 2:** Disease knowledge, medical experience, and expenditure in patients with early-onset and late-onset colorectal cancer

	Overall (*N* = 4572)	EOCRC, < 50 years (*N* = 991)	LOCRC, ≥ 50 years (*N* = 3581)	*P* value
**Awareness of CRC risk factors, yes**	1591 (34.9)	366 (37.1)	1225 (34.3)	0.107
**Awareness of CRC screening, yes**	689 (15.1)	169 (17.1)	520 (14.6)	0.055
**Awareness of CRC treatment, yes**	2019 (44.2)	441 (44.5)	1578 (44.1)	0.827
**Undergoing the colonoscopy before the first diagnosis, yes**	120 (2.6)	29 (2.9)	91 (2.5)	0.575
**Barriers to undergo colonoscopy**				
Lack of awareness, yes	3868 (87.0)	834 (86.8)	3034 (87.1)	0.865
No time for a colonoscopy, yes	368 (8.3)	93 (9.7)	275 (7.9)	0.087
Heard that colonoscopy is a painful procedure, yes	716 (16.1)	141 (14.7)	575 (16.5)	0.189
The cost of colonoscopy is high, yes	172 (3.9)	31 (3.2)	141 (4.0)	0.258
Waiting in line for colonoscopy appointment, yes	172 (3.9)	42 (4.4)	130 (3.7)	0.414
Insurance doesn’t cover it, yes	97 (2.2)	22 (2.3)	75 (2.2)	0.803
**Undergoing gene testing, including* RAS***, ***BRAF***, **and MSI, yes**	1974 (47.1)	518 (56.7)	1456 (44.4)	< 0.001
**Barriers to undergo gene testing**				0.052
Target therapy is not accepted (other treatment options are considered to be sufficient)	357 (16.1)	45 (11.3)	312 (17.2)	
The test is too expensive and not reimbursable	528 (23.8)	107 (26.8)	421 (23.2)	
Anxious to receive treatment and unwilling to wait for genetic test results	125 (5.6)	18 (4.5)	107 (5.9)	
Plan to blind-eat targeted drugs	32 (1.4)	6 (1.5)	26 (1.4)	
Lack of knowledge	952 (43.0)	183 (45.9)	769 (42.3)	
Others	222 (10.0)	40 (10.0)	182 (10.0)	
***RAS*** ** mutation, yes**	275 (32.5)	59 (29.4)	216 (33.5)	0.301
***BRAF*** ** mutation, yes**	79 (9.3)	23 (11.4)	56 (8.7)	0.266
**MSI, yes**	49 (5.8)	13 (6.5)	36 (5.6)	0.608
**Undergoing the targeted therapy, yes**	1442 (31.7)	364 (36.8)	1078 (30.3)	< 0.001
**Barriers to undergo the targeted therapy**				
The physician did not mention it to patients, yes	1253 (40.4)	266 (42.6)	987 (39.8)	0.231
Genetic tests identify tumors that will not respond to targeted therapy, yes	203 (6.5)	40 (6.4)	163 (6.6)	0.944
There is no confidence in the efficacy of these targeted drug treatments, yes	703 (22.7)	102 (16.3)	601 (24.3)	< 0.001
Cannot afford the cost of medical treatment, yes	736 (23.7)	143 (22.9)	593 (23.9)	0.618
**Treatments**				
Surgery, yes	3823 (83.8)	836 (84.4)	2987 (83.6)	0.612
Endoscopic treatment, yes	141 (3.1)	31 (3.1)	110 (3.1)	0.999
Radiotherapy, yes	1001 (21.9)	266 (26.8)	735 (20.6)	< 0.001
Chemotherapy, yes	3943 (86.4)	906 (91.4)	3037 (85.0)	< 0.001
Immunotherapy, yes	108 (2.4)	24 (2.4)	84 (2.4)	0.992
**Out-of-pocket medical expenditure, Chinese Yuan**				< 0.001
< 50,000	1147 (25.2)	187 (18.9)	960 (26.9)	
50,000-100,000	1859 (40.8)	365 (36.9)	1494 (41.8)	
100,000-200,000	1040 (22.8)	259 (26.2)	781 (21.9)	
≥ 200,000	514 (11.3)	179 (18.1)	335 (9.4)	
**Medical expenditure reimbursement ratio (%)**	0.59 (0.18)	0.56 (0.18)	0.59 (0.18)	< 0.001
**Annual household income, Chinese Yuan**				< 0.001
None	759 (16.7)	106 (10.7)	653 (18.3)	
< 50,000	1855 (40.7)	353 (35.7)	1502 (42.1)	
50,000-100,000	1289 (28.3)	309 (31.3)	980 (27.5)	
100,000-200,000	521 (11.4)	163 (16.5)	358 (10.0)	
≥ 200,000	132 (2.9)	57 (5.8)	75 (2.1)	
**Cost of colorectal cancer treatment from the perspective of patients**				0.413
< 50,000	1314 (28.9)	273 (27.7)	1041 (29.2)	
50,000-100,000	1510 (33.2)	319 (32.3)	1191 (33.5)	
100,000-200,000	1103 (24.3)	245 (24.8)	858 (24.1)	
200,000-500,000	524 (11.5)	124 (12.6)	400 (11.2)	
≥ 500,000	96 (2.1)	26 (2.6)	70 (2.0)	
**Health insurance**				0.005
Urban basic medical insurance	1919 (42.0)	379 (38.2)	1540 (43.0)	
Urban basic medical insurance	981 (21.5)	205 (20.7)	776 (21.7)	
New rural cooperative medical scheme	1552 (33.9)	373 (37.6)	1179 (32.9)	
Other	120 (2.6)	34 (3.4)	86 (2.4)	

*Abbreviations* EOCRC, Early onset colorectal cancer; LOCRC, Late onset colorectal cancer; CRC, colorectal cancer; MSI, microsatellite instability

Values are presented as mean (standard deviations) for continuous variables or n (%) for categorical variables

Table 3 presents the HRQOL scores for patients with EOCRC and LOCRC. Compared with the LOCRC group, the EOCRC group did not exhibit a significant difference in the overall HRQOL score (EOCRC = 127.63; LOCRC = 128.33; *P* = 0.441). However, concerning the subscales, the EOCRC group had a notably higher score in the physical scale of EORTC QLQ-C30 when compared to the LOCRC group, and it also experienced a more significant financial impact. No significant differences were observed in the scores of other subscales between the EOCRC and LOCRC groups. In patients with CRC, multivariate analysis demonstrates that there is no significant association between early diagnosis of CRC and HRQOL (beta: −0.753, *P* value: 0.307). The estimated effects of the regression analysis are presented in Table S4.

**Table 3:** Health-related quality of life in patients with early-onset and late-onset colorectal cancer

	Number of items	Overall (*N* = 4572)	EOCRC, < 50 years *(N* = 991)	LOCRC, ≥ 50 years (*N* = 3705)	*P* value
**Overall HRQOL** ^*^		128.18 (24.72)	127.63 (24.52)	128.33 (24.77)	0.441
**FACT-C** ^**¶**^	**36**				
Physical well-being	10	31.53 (5.85)	31.25 (6.12)	31.61 (5.77)	0.082
Social/Family well-being	7	22.98 (5.69)	23.04 (5.61)	22.97 (5.71)	0.722
Emotional well-being	5	14.89 (4.33)	14.75 (4.49)	14.92 (4.28)	0.262
Functional well-being	7	14.69 (6.95)	14.85 (6.84)	14.65 (6.98)	0.410
Colorectal cancer subscale	7	17.79 (4.55)	17.90 (4.48)	17.76 (4.57)	0.368
**EORTC QLQ-C30**	**9**				
**Functional scales and/or items** ^**¶**^					
Physical	1	3.44 (1.02)	3.60 (0.90)	3.39 (1.05)	< 0.001
Cognitive	1	3.10 (1.00)	3.10 (1.03)	3.11 (0.99)	0.924
Emotional	2	6.24 (1.79)	6.05 (1.91)	6.30 (1.76)	< 0.001
Social	2	5.25 (2.20)	5.04 (2.30)	5.31 (2.17)	0.001
**Symptom items** ^**§**^					
Fatigue	1	0.89 (1.05)	0.83 (1.03)	0.90 (1.05)	0.051
Sleep disturbance	1	1.15 (1.16)	1.18 (1.19)	1.14 (1.16)	0.349
Financial impacts	1	1.58 (1.24)	1.79 (1.29)	1.52 (1.22)	< 0.001

Values are presented as mean (standard deviations)

^*^A higher score indicates a better quality of life

^¶^A higher score indicates a higher level of functioning

^§^A higher score indicates a greater degree of symptoms

## Discussion

Based on this multicenter, cross-sectional study, we conducted a comprehensive evaluation of the clinical profiles of both EOCRC and LOCRC patients in China. We found that EOCRC patients generally had higher education levels and a higher incidence of widespread metastases. Additionally, they were more prone to undergo gene testing and opt for aggressive treatments like targeted therapy, radiotherapy, and chemotherapy. Despite these differences, their HRQOL was similar to that of LOCRC patients, a finding that merits further investigation into the contributing factors. These unique characteristics of EOCRC underscore the necessity of a thorough evaluation of patient subgroups and indicate a need for tailored screening strategies and treatment protocols, especially in younger demographics.

In this cross-sectional study, we found that EOCRC patients exhibited higher levels of education, greater household income, and a more comprehensive understanding of CRC in comparison to LOCRC patients. This finding may be attributed to the age difference between EOCRC and LOCRC patients. The EOCRC group, comprising younger patients, tended to have received higher levels of education, reflecting the evolving education landscape in China. Patients with higher education levels tended to have higher incomes and more disease knowledge.

Our results revealed that the EOCRC group tends to exhibit more widespread metastases and a more advanced TNM stage than the LOCRC group, though this difference in the TNM stage did not reach statistical significance. Our findings were consistent with previous studies in the Western population and Chinese, which showed that the EOCRC group had a higher risk of lymph node metastases [18] compared to the LOCRC group. A previous study revealed that the advanced TNM stage at diagnosis in EOCRC patients does not seem to be explained simply by the longer time to diagnosis, suggesting that biological factors may be important determinants of the TNM stage at diagnosis [19]. Although tumor biology may be an important determinant of the TNM stage at diagnosis, clinicians need to recognize CRC alarm symptoms, family history, and genetic syndromes, to speed evaluation and diagnosis of younger patients and potentially improve outcomes.

We also found that patients with EOCRC were more likely to receive gene testing, perioperative chemoradiotherapy, and targeted therapy than those with LOCRC while experiencing similar benefits in HRQOL. Treatment recommendations for patients with EOCRC and LOCRC are consistent with major clinical practice guidelines [20, 21]. However, we found more intensive treatment among young patients. This finding was consistent with previous studies indicating that young patients with CRC received more aggressive surgical treatment, greater resection extent, and more perioperative chemoradiotherapy and targeted therapy than those with LOCRC [7, 22–24]. Nevertheless, whether these aggressive treatments would lead to survival benefits remains controversial. Several studies report a worse prognosis, while others demonstrate equivalent or superior outcomes among younger patients [22, 24–26]. In our study, we found that, despite being more likely to receive chemoradiotherapy and chemotherapy, younger patients had a comparable HRQOL to their older counterparts. This finding can be attributed to two contrasting factors: the inherently more aggressive clinicopathological features and advanced TNM stage of EOCRC, and the more intensive treatment it received. These factors together contributed to similar well-being between the EOCRC and LOCRC groups.

Our study conducted a nationwide survey to gather a representative sample of the Chinese population, which ensures the generalizability of our findings across diverse demographic groups. Additionally, we systematically identified distinctive features of EOCRC by comparing a range of characteristics, including demographics, clinical features, disease knowledge, medical experiences, expenditures, and health-related quality of life. Our study underscored the necessity for tailored screening and treatment strategies for younger CRC patients, offering significant insights that could influence future public health policies and clinical practices in China.

Our study has several limitations. Firstly, we cannot compare the characteristics of EOCRC stratified by predisposing conditions. However, previous studies have indicated that only a minority of EOCRC cases are attributable to hereditary syndromes [2, 27], suggesting that this minority may not significantly impact our findings. Secondly, the self-reported data, including disease knowledge, CRC screening, and HRQOL, may be susceptible to recall biases. Nevertheless, we have taken several measures to maintain the accuracy of the data [28, 29]. These included formulating clear and precise questions to reduce variation in comprehension and ensuring the collection of valid and reliable data. Moreover, in-person interviews have been employed to facilitate more accurate recall data. Finally, we did not collect the personal oncological history, family history of diseases and comprehensive comorbidities, which may have influence on HROQL.

## Conclusion

Our study found that EOCRC patients, despite having a higher prevalence of widespread metastases and receiving more aggressive treatment and gene testing, still exhibited an HRQOL similar to that of the LOCRC group.

### Electronic supplementary material

Below is the link to the electronic supplementary material.

Supplementary Material 1

## Data Availability

Data are available upon reasonable request to the corresponding authors.
