# Supplementary material for: Comparative characteristics of early-onset vs. late-onset advanced colorectal cancer: a nationwide study in China
Source: BMC Cancer. 2024 Apr 20;24:503. doi: 10.1186/s12885-024-12278-7 (PMC11031847; doi:10.1186/s12885-024-12278-7)
Supplement: Supplementary file 1 — Supplementary Material 1 [file 12885_2024_12278_MOESM1_ESM.docx]

**Supplemental materials**


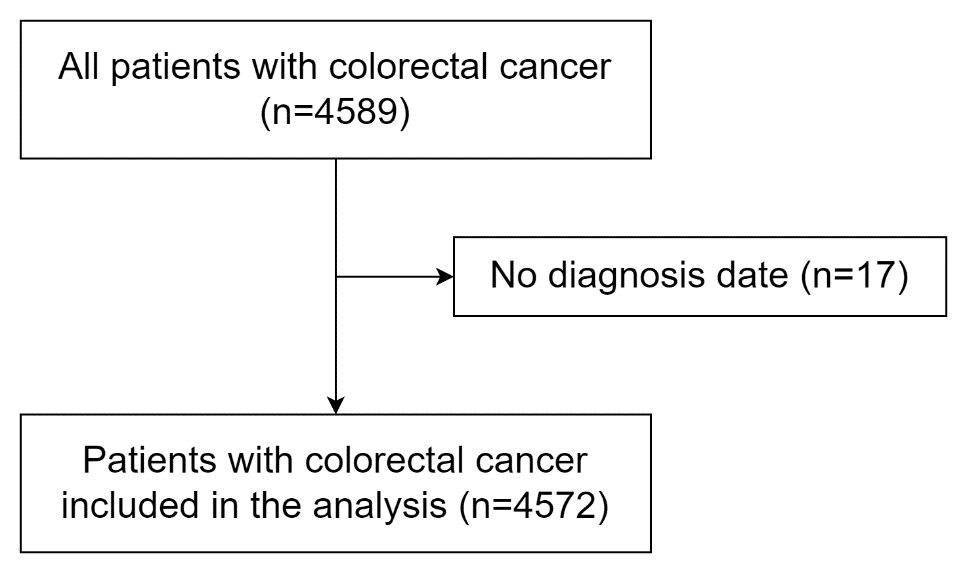


**Figure S1** Flow chart of patient selection process.


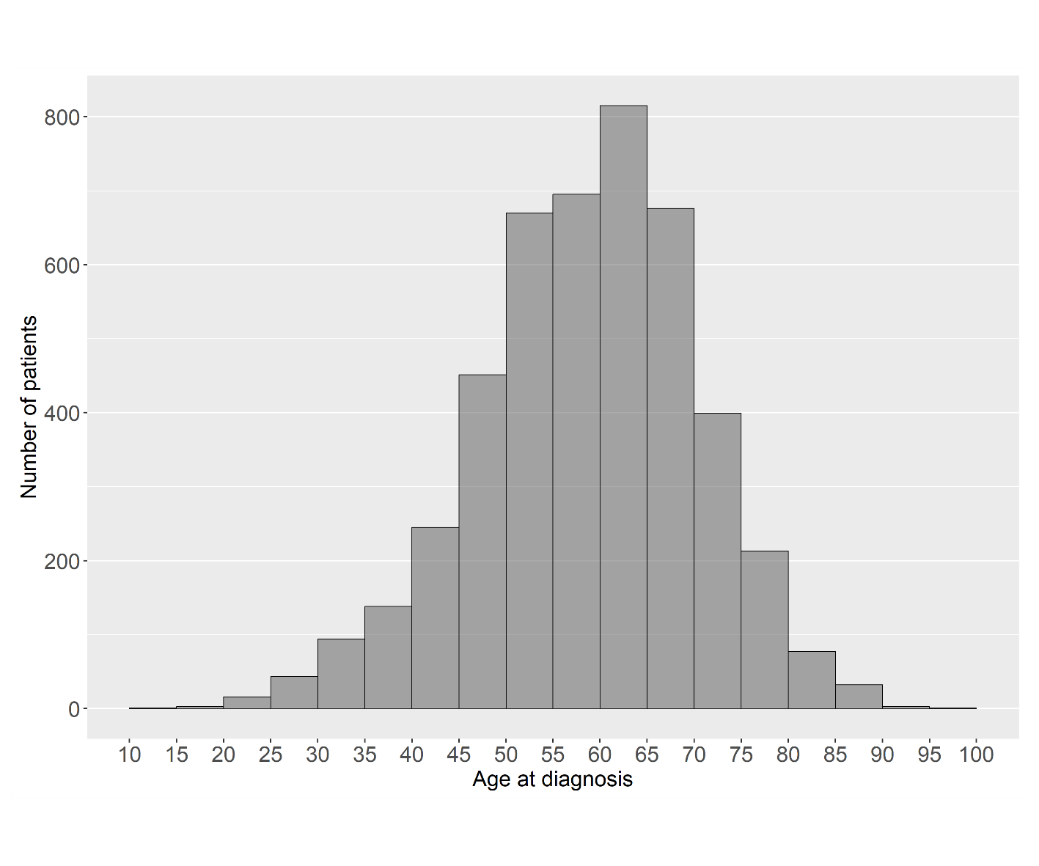


Figure S2 Distribution of age at diagnosis of the included 4572 cases of colorectal cancer

Table S1 Distribution of sample size across geographical regions.

| Geographical regions | Hospitals | Sample Size |
| --- | --- | --- |
| Eastern | ⑪ Zhejiang Cancer Hospital | 435 |
|  | ⑩ School of Nursing, Jining Medical University | 877 |
| Northern | ③ Beijing Office for Cancer Prevention and Control, Peking University Cancer Hospital & Institute | 288 |
|  | ④ Department of Public Health and Preventive Medicine, Baotou Medical College | 275 |
| Southern | ⑫ Sun Yat-sen University Cancer Center | 168 |
|  | ⑬ The First Affiliated Hospital, Jinan University | 165 |
|  | ⑭ The First Affiliated Hospital of Guangxi Medical University | 163 |
|  | ⑮ Wuzhou Red Cross Hospital | 169 |
| Central | ⑧ Henan Cancer Hospital | 331 |
|  | ⑨ Xinxiang Central Hospital | 358 |
| Northeast | ① Cancer Hospital of China Medical University, Liaoning Cancer Hospital and Institute | 173 |
|  | ② Dalian Medical University | 191 |
| Southwest | ⑱ Sichuan Cancer Hospital & Institute | 222 |
|  | ⑲ Chengdu Medical College | 102 |
|  | ⑯ Chongqing Cancer Hospital | 164 |
|  | ⑰ Chongqing Medical University | 164 |
| Northwest | ⑦ Affiliated Tumor Hospital, Xinjiang Medical University | 166 |
|  | ⑤ Gansu Provincial Cancer Hospital | 88 |
|  | ⑥ The First People's Hospital of Lanzhou City | 73 |

Table S2 Semi-structured questionnaire on CRC knowledge

| Questions | Answers |
| --- | --- |
| 1. Before you were diagnosed with CRC, did you think which of the following was/were the high- risk factors of CRC? | A. Aged 50~74. |
|  | B. A history of colorectal adenoma. |
|  | C. A history of chronic diarrhoea, chronic constipation or bloody stool. |
|  | D. A history of chronic appendicitis or appendectomy. |
|  | E. A history of chronic cholecystitis or cholecystectomy. |
|  | F. Lack of physical exercise. |
|  | G. Unhealthy habits such as heavy smoking or drinking. |
|  | H. Unhealthy diet such as excessive intake of red meat, or less intake of vegetables and cellulose. |
|  | I. A first- degree family history of CRC. |
|  | J. Others, please specify: _____. |
|  | K. I did not know. |
| 2. Before you were diagnosed with CRC, did you think which of the following was/were the procedure of CRC screening? | A. For the general population aged 50~74, questionnaire survey is needed for the first screening, then decide whether colonoscopy is necessary. |
|  | B. For the general population aged 50~74, faecal occult blood test should be done at least once a year. If it is positive, colonoscopy is required. |
|  | C. For the general population aged 50~74, colonoscopy screening should be done at least once every 5 years. |
|  | D. For a high- risk population, colonoscopy screening should be done at least once every year. |
|  | E. Others, please specify: ________. |
|  | F. I did not know. |
| 3. Before you were diagnosed with CRC, did you think which of the following was/were the treatment methods of CRC? | A. Endoscopic treatment (endoscopic mucosal resection). |
|  | B. Surgical treatment (colectomy + regional lymphadenectomy). |
|  | C. Radiotherapy. |
|  | D. Chemotherapy. |
|  | E. Targeted therapy. |
|  | F. Traditional Chinese medicine treatment. |
|  | G. I did not know. |

Table S3 Selected nine items from EORTC QLQ-C30

| **Scales or items** | **Questions** |
| --- | --- |
| Physical scale | Do you need help with eating, dressing, washing yourself or using the toilet? |
| Cognitive scale | Have you had difficulty remembering things? |
| Emotional function | Did you feel irritable? |
|  | Did you feel depressed? |
| Social function | Has your physical condition or medical treatment interfered with your family life? |
|  | Has your physical condition or medical treatment interfered with your social activities? |
| Fatigue | Were you tired? |
| Sleep disturbance | Have you had trouble sleeping? |
| Financial impacts | Has your physical condition or medical treatment interfered with your financial difficulties? |

Table S4 The estimated effects of multivariate regressions on early-onset CRC and HRQOL.

| Variables | Beta | Se | *P* value |
| --- | --- | --- | --- |
| Early-onset CRC | -0.753 | 0.737 | 0.307 |
| HRQOL before treatment | 0.614 | 0.012 | < 0.001 |
| Sex |  |  |  |
| Male | 2.996 | 0.614 | < 0.001 |
| Female | Reference | | |
| Clinical stage at first diagnosis |  |  |  |
| Ⅰ | Reference | | |
| Ⅱ | -1.066 | 2.008 | 0.596 |
| Ⅲ | -1.406 | 1.933 | 0.467 |
| Ⅳ | -4.800 | 1.948 | 0.014 |
| Location of cancer |  |  |  |
| Colon | Reference | | |
| Rectum | -3.841 | 0.602 | < 0.001 |
| Education level |  |  |  |
| Primary school or below | Reference | | |
| Middle school | 0.016 | 0.770 | 0.983 |
| High school/specialized secondary schools | 0.122 | 0.840 | 0.884 |
| University/specialty or above | 1.490 | 0.956 | 0.119 |

CRC, colorectal cancer; HRQOL, health-related quality of life; LOCRC, late-onset colorectal cancer; EOCRC, early-onset colorectal cancer.
